# RAFusion: Integrating Residual Attention for Global Context-Aware 6D Pose Estimation

**DOI:** 10.3390/s26144571

**Published:** 2026-07-19

**Authors:** Wenjin Zhao, Yi Lai, Qixian Zhang, Kang An, Hui Zhang

**Affiliations:** 1 College of Information, Mechanical and Electrical Engineering, Shanghai Normal University, Shanghai 201418, China; 1000555097@smail.shnu.edu.cn (W.Z.); willow984@outlook.com (Y.L.); ankang@shnu.edu.cn (K.A.); 2 School of Computer Science and Technology, Tongji University, Shanghai 201804, China; zhangqx@tongji.edu.cn

**Keywords:** 6D pose estimation, RGB-D perception, residual attention, feature fusion

## Abstract

6D object pose estimation from RGB-D data is a core problem in robotic perception. Representative approaches such as DenseFusion fuse per-pixel RGB and depth features, yet they still face challenges under heavy occlusion and in cluttered backgrounds. This paper revisits the DenseFusion architecture from the perspective of feature enhancement and global context modeling and proposes an enhanced network named RAFusion, which strengthens feature representation through two complementary modules. First, Squeeze-and-Excitation (SE) blocks are integrated into the RGB feature extractor to adaptively reweight channel responses, enhancing salient appearance cues while suppressing noise. Second, a RealFormer-based block is introduced before global pooling, applying residual self-attention to the fused RGB-D features for more effective global context modeling. Experiments on the LINEMOD and Occlusion LINEMOD datasets show that RAFusion improves the DenseFusion baseline and achieves competitive performance compared with representative 6D pose estimation methods. Ablation studies and computational analysis further support the effectiveness of the proposed modules.

## 1. Introduction

6D object pose estimation, which aims to determine the precise 3D rotation and translation of an object with respect to a camera, is a fundamental problem in computer vision and robotics [1,2,3]. It serves as a prerequisite for many applications, including robotic manipulation, augmented reality, autonomous driving, action recognition [4], and image retrieval [5], where intelligent interaction with the environment is essential [6,7,8]. With the increasing availability of low-cost depth sensors, RGB-D-based methods have become the dominant paradigm. These methods leverage the complementary nature of color and geometry: RGB data provides rich texture information, while depth data encodes spatial structure, a capability that is also crucial for visual SLAM in dynamic environments [9]. Consequently, RGB-D-based methods generally achieve improved accuracy and robustness compared with RGB-only approaches [10,11].

DenseFusion [12] is a pioneering pixel-wise fusion architecture that effectively combines RGB and depth features. However, its default design choices restrict performance under heavy occlusion and clutter. First, the standard convolutional backbone lacks input-adaptive channel recalibration, which may limit the suppression of channel responses dominated by occluders or background clutter [13]. Second, its reliance on global pooling for context aggregation limits its ability to capture long-range dependencies, which are critical for inferring the holistic object structure [14,15,16].

To tackle these issues, we propose RAFusion, which revisits the DenseFusion architecture with two targeted enhancements. RAFusion targets two complementary limitations of DenseFusion-style RGB-D pose estimation: the lack of explicit input-adaptive channel recalibration before RGB-D fusion and limited interaction modeling among fused point-pixel features before global aggregation. First, Squeeze-and-Excitation (SE) blocks [13] are incorporated into the RGB encoder to adaptively suppress occlusion-induced noise through channel-wise recalibration. Second, a RealFormer-based module [15] is inserted before global pooling in the fusion stage, using residual self-attention to model long-range dependencies among fused point-pixel features. The main contributions of this paper are as follows:

1. SE-Enhanced Color Feature Extractor. The proposed RAFusion network augments the RGB branch of a DenseFusion-style baseline with Squeeze-and-Excitation blocks. These blocks adaptively reweight channel responses, improving the expressiveness of appearance features in cluttered and partially occluded scenes.
2. RealFormer-Based Global Fusion Module. A RealFormer-style residual attention module is incorporated into the RGB-D fusion stage to capture long-range dependencies in the fused representation. The module refines the fused feature sequence before average pooling, and its computational characteristics are evaluated in Section 4.4.2.
3. Comprehensive Empirical Validation. The RAFusion network is evaluated on the LINEMOD and Occlusion LINEMOD datasets. The experiments include comparisons with DenseFusion and representative methods, component ablations, stochastic point-sampling sensitivity, qualitative success and failure cases, and computational efficiency analysis.

## 2. Related Work

### 2.1. RGB-D-Based 6D Pose Estimation

Current RGB-D methods for 6D pose estimation can be broadly categorized into correspondence-based and dense-fusion-based approaches. RGB-based methods estimate pose from a query RGB image together with method-specific references, templates, or object representations. RGB-D methods additionally use metric depth geometry from the test scene. The comparative tables therefore group RGB-based and RGB-D methods separately according to their test-time input modalities.

Correspondence-based methods establish 2D-3D matches to solve for pose via PnP algorithms [17]. While early works using hand-crafted features like SIFT [18], SURF [19], and ORB [20] struggled with textureless objects, deep learning has enabled robust descriptor learning [21,22] and direct coordinate prediction. For instance, PVNet [23] and PVN3D [11] employ voting networks to localize 2D keypoints and 3D offsets, respectively, while DPOD [24] predicts dense coordinate maps. Although accurate, these multi-stage pipelines often incur high computational costs due to optimization steps like RANSAC [25].

Dense-fusion-based methods provide an efficient end-to-end alternative. DenseFusion [12] extracts RGB and depth features separately using CNNs and PointNets, fusing them pixel-wise for direct pose prediction. Subsequent works have extended this paradigm to volumetric fusion [26] and coarse-to-fine localization [27]. However, most such methods rely on standard global pooling for context aggregation. This operation compresses spatial details and fails to capture long-range dependencies crucial for handling heavy occlusion, a limitation addressed in this work.

### 2.2. Feature Enhancement and Attention Mechanisms

Attention mechanisms have become a cornerstone in modern computer vision, enabling networks to focus on the most informative parts of the input [28]. Channel attention, epitomized by the Squeeze-and-Excitation (SE) Network [13,29], adaptively recalibrates channel-wise feature responses. It has proven highly effective in boosting feature discriminability for tasks ranging from image classification to object detection [30]. In the context of 6D pose estimation, where occlusion often introduces noise and ambiguity into feature maps, channel attention offers a powerful tool to suppress irrelevant background signals [31]. Recent works have begun to explore attention in this domain; for example, 3DFeat-Net [32] uses attention to learn robust 3D feature detectors. By integrating SE blocks into its color branch, the proposed RAFusion enables the subsequent fusion module to obtain cleaner and more object-centric appearance features.

### 2.3. Global Context Modeling

Capturing global context is important when local cues are ambiguous. However, standard global average pooling [12] or max pooling can discard spatial information.

Recently, Transformers [14,33] and self-attention mechanisms have demonstrated a strong capacity for modeling long-range dependencies. In the 3D domain, Point Transformer-style architectures further demonstrate the effectiveness of self-attention in point cloud processing [34,35]. However, training deep Transformers on limited pose data can be unstable. The RealFormer [15] architecture addresses this issue by introducing a residual-attention mechanism, allowing attention scores to be accumulated and refined across layers. This residual design helps preserve structural information across layers. By incorporating a RealFormer-based module, RAFusion constructs a global feature representation that connects distant object parts and supports pose inference under severe occlusion. Residual learning has also been used in other domains to approximate complex nonlinear mappings. ResidualJointAntennaNet [36] employs deep feed-forward residual blocks, while RAFusion accumulates pre-softmax attention logits across layers. These different residual formulations provide complementary perspectives for designing deeper pose-estimation architectures.

## 3. Methods

### 3.1. Overall Framework

**Figure 1:**
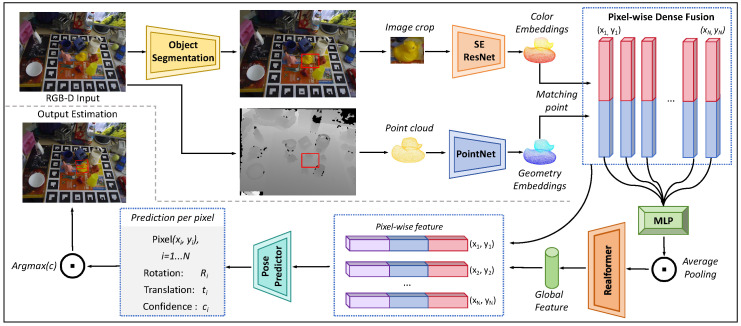
The overall architecture of the proposed RAFusion network. The pipeline fuses RGB and depth features extracted by an SE-ResNet and a PointNet, respectively. A RealFormer module is inserted between pixel-wise fusion and average pooling; the pooled global context is concatenated with context-enhanced point features for pose and confidence prediction. Red squares mark the corresponding target region in the RGB and depth views, colored feature bars distinguish color and geometry embeddings, and arrows indicate the direction of data flow.

As shown in Figure 1, the RAFusion pipeline first segments the target object to obtain a cropped RGB image and a masked point cloud. Parallel SE-ResNet and PointNet branches extract color and geometric embeddings, which are fused at corresponding point-pixel locations. The fused sequence is then refined by the RealFormer block before average pooling. Average pooling of the context-enhanced sequence produces a global vector, which is concatenated with each enhanced per-point feature for pose and confidence prediction. The pose with the highest confidence is selected and can subsequently be processed by the iterative refinement network.

### 3.2. Adaptive Color Feature Extraction

In 6D pose estimation, standard convolutional backbones lack an explicit mechanism for input-adaptive channel recalibration, so channels dominated by occluders or background clutter may remain influential [37]. To address this, RAFusion integrates Squeeze-and-Excitation blocks into the color encoder. The RGB branch uses a ResNet-18-style encoder with pyramid scene parsing upsampling. An SE unit is inserted into every residual block after the second 3 by 3 convolution and before residual addition, so the residual response is channel-recalibrated before it is merged with the skip connection. By modeling inter-channel dependencies, the SE mechanism amplifies salient object features and suppresses background noise. The computational overhead introduced by the SE units is quantified in Section 4.4.2.

**Figure 2:**
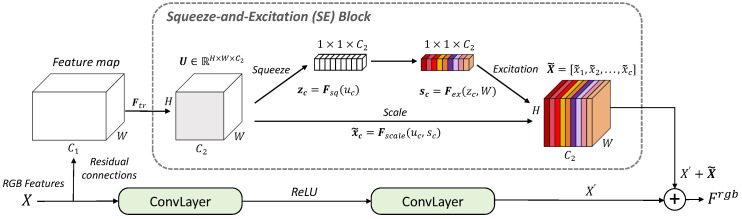
SE-enhanced residual block for adaptive color feature extraction. In each ResNet-18 residual block, the SE operation is applied after the second convolution and before residual addition. The SE block computes channel-wise weights s, and the recalibrated residual features $\tilde{X}$ are combined with the skip connection to produce $F^{rgb}$. Colored bars depict channel responses and channel weights, arrows indicate feature flow, and the circled plus sign denotes residual addition.

As illustrated in Figure 2, let $U\in R^{H\times W\times C}$ denote the feature map output by a convolutional transformation $F_{tr}$. The SE block recalibrates U through three key operations: Squeeze, Excitation, and Scale. First, the Squeeze operation $F_{sq}$ uses global average pooling to compress spatial information into a channel descriptor $z\in R^{C}$. The *c*-th element is

(1) $$z_{c}=F_{sq}\left(u_{c}\right)=\frac{1}{H\times W}\sum_{i=1}^{H}\sum_{j=1}^{W}u_{c}\left(i,j\right),$$

where $u_{c}\left(i,j\right)$ represents the value at position (i,j) of the *c*-th channel. This operation embeds the global distribution of feature responses, allowing the network to perceive the image’s global context.

Next, the Excitation operation $F_{ex}$ captures channel-wise dependencies. To fully utilize the aggregated information, a simple gating mechanism with a sigmoid activation is employed:

(2) $$s=F_{ex}\left(z,W\right)=\sigma \left(W_{2}\delta \left(W_{1}z\right)\right),$$

where δ refers to the ReLU activation function, σ is the Sigmoid function, $W_{1}\in R^{\frac{C}{r}\times C}$ and $W_{2}\in R^{C\times \frac{C}{r}}$ are weights of two fully connected layers, and *r* is the reduction ratio. This structure learns non-linear channel interactions to identify the most informative features for pose estimation.

Finally, the Scale operation $F_{scale}$ performs the adaptive reweighting. The original feature map U is recalibrated by the learned channel weights s:

(3) $${\tilde{x}}_{c}=F_{scale}\left(u_{c},s_{c}\right)=s_{c}\cdot u_{c},$$

where $\tilde{X}=\left[{\tilde{x}}_{1},{\tilde{x}}_{2},\ldots ,{\tilde{x}}_{C}\right]$ is the final output of the SE block.

Through this process, Equation (3) performs soft channel-wise feature recalibration. Each coefficient $s_{c}$ scales the complete *c*-th feature map. In heavily occluded scenes, feature channels dominated by occluder or background responses may receive smaller weights, whereas channels carrying more discriminative object-related responses may be emphasized, improving the selectivity of the RGB representation.

### 3.3. Residual Attention for Global Context Modeling

After pixel-wise fusion of color and geometry embeddings, DenseFusion directly applies average pooling to obtain a global feature, without explicitly modeling interactions among distant visible regions. RAFusion applies residual self-attention to the fused point-pixel sequence before global aggregation and then averages the context-enhanced sequence to obtain the global vector. The resulting global representation is concatenated with the enhanced per-point features for pose prediction. Unlike standard Transformers, RealFormer propagates and accumulates attention logits across layers, preserving structural information and modeling global dependencies more effectively than pooling alone.

Let $X\in R^{N\times d}$ be the input sequence of fused point-pixel features, where *N* is the number of points and *d* is the feature dimension. A trainable absolute positional embedding matrix $P\in R^{N\times d}$ is added element-wise to the sampled-point sequence before the first RealFormer layer, yielding $X_{0}=X+P$. Each sampled sequence position therefore has one learned *d*-dimensional embedding. In a standard Transformer layer, the attention matrix A is typically computed solely from the current layer’s queries Q and keys K. RealFormer instead adds the pre-softmax attention logits from the previous layer to the current logits, forming a residual connection within the attention mechanism:

(4) $$S_{curr}=\frac{QK^{T}}{\sqrt{d_{k}}}+S_{prev},\;A_{curr}=\mathrm{Softmax}\left(S_{curr}\right).$$

Here, $d_{k}$ denotes the dimensionality of the query and key vectors in each attention head. This cumulative attention score $A_{curr}$ is then used to weight the values V:

(5) $$\mathrm{Attention}\left(Q,K,V\right)=A_{curr}V.$$

**Figure 3:**
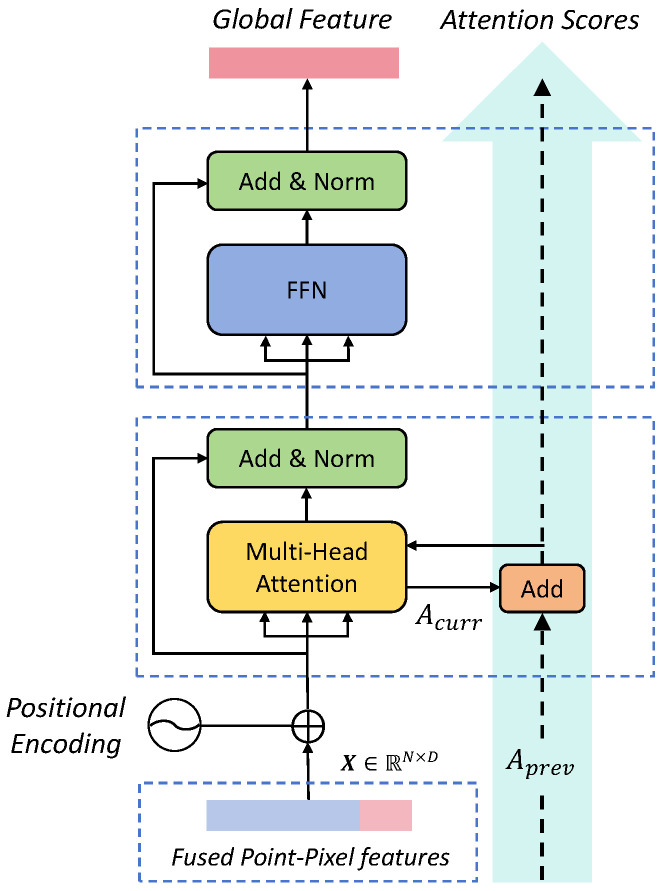
Structure of the RealFormer-based global fusion module. Positionally encoded features X pass through a Transformer layer in which the residual-attention path adds the previous pre-softmax logits $S_{\ell -1}$ to the current logits $S_{\ell }$. The resulting attention weights $A_{\ell }$ support the accumulation of global context across layers. Colored boxes denote processing blocks, solid arrows show feature flow, and the vertical dashed arrow indicates the residual attention-score path.

The output is then processed by a standard Feed-Forward Network (FFN) with Add & Norm layers, as shown in Figure 3.

This residual design offers two key benefits: it accumulates attention scores to capture stable long-range relationships across layers, and it preserves critical early-layer structural information.

After the final RealFormer layer, average pooling is applied to the context-enhanced sequence $X_{L}$ to obtain the global vector g. The vector g is concatenated with each enhanced feature $X_{L}^{\left(i\right)}$, producing ${\tilde{f}}_{i}=\left[X_{L}^{\left(i\right)},g\right]$ for pose and confidence prediction. The complete workflow is detailed in Algorithm 1.
**Algorithm 1** RealFormer Residual Attention for Global Context Modeling**Require:** Per-point fused features $X\in R^{N\times d}$, RealFormer depth *L*, positional encoding P
**Ensure:** Global context vector $g\in R^{d_{g}}$ and enriched features ${\left\{{\tilde{f}}_{i}\right\}}_{i=1}^{N}$
1:$X_{0}\leftarrow X+P$,   $S_{-1}\leftarrow 0$2:**for** ℓ=0 to L−1 **do**3:      $Q_{\ell }\leftarrow X_{\ell }W_{\ell }^{Q}$4:      $K_{\ell }\leftarrow X_{\ell }W_{\ell }^{K}$5:      $V_{\ell }\leftarrow X_{\ell }W_{\ell }^{V}$6:      $S_{\ell }\leftarrow \frac{Q_{\ell }K_{\ell }^{T}}{\sqrt{d_{k}}}+S_{\ell -1}$7:      $A_{\ell }\leftarrow \mathrm{Softmax}\left(S_{\ell }\right)$8:      $Y_{\ell }\leftarrow A_{\ell }V_{\ell }$9:      $X_{\ell }^{\prime }\leftarrow \mathrm{AddNorm}\left(X_{\ell },Y_{\ell }\right)$10:     $X_{\ell +1}\leftarrow \mathrm{AddNorm}\left(X_{\ell }^{\prime },\mathrm{FFN}\left(X_{\ell }^{\prime }\right)\right)$11:**end for**12:$g\leftarrow \frac{1}{N}\sum_{i=1}^{N}X_{L}^{\left(i\right)}$13:**for** i=1 to *N* **do**14:     ${\tilde{f}}_{i}\leftarrow \left[X_{L}^{\left(i\right)},\;g\right]$15:**end for**16:**return** $g,\;{\left\{{\tilde{f}}_{i}\right\}}_{i=1}^{N}$


## 4. Results and Discussion

### 4.1. Implementation Details and Datasets

All training and accuracy experiments were implemented using Python 3.8 and PyTorch 2.7.0+cu128 on NVIDIA A100 GPUs (NVIDIA Corporation, Santa Clara, CA, USA) running Ubuntu 20.04 and CUDA 12.8. RAFusion follows the DenseFusion training configuration [12]. The model is trained with a batch size of 16 and an initial learning rate of 0.0001. The confidence regularization coefficient is set to 0.013 and multiplies the negative log-confidence term in the pose loss.

The RGB branch is based on SE-ResNet-18 with pyramid scene parsing upsampling. The depth branch follows the DenseFusion PointNet-style encoder: shared 1×1 point convolutions map the 3D coordinates from 3 to 64 and then 128 channels; the aligned 32-channel RGB embedding is mapped to 64 and then 128 channels; and the intermediate geometry and appearance features are concatenated to form 128- and 256-channel point-wise representations, followed by shared 1×1 convolutions from 256 to 512 and then 1024 channels. ReLU activations are used between these layers. Each target is represented by N=500 sampled points, and the fused feature dimension entering RealFormer is d=1024. Unless otherwise stated in the sensitivity study, RealFormer uses two layers, eight attention heads, a 2048-dimensional FFN hidden layer (expansion factor 2), dropout of 0.1, and the trainable positional matrix $P\in R^{500\times 1024}$ defined in Section 3.3. The pose refinement network and its use are kept identical for DenseFusion and RAFusion.

Experiments use two standard benchmarks: LINEMOD, containing RGB-D sequences of 13 textureless objects, and Occlusion LINEMOD, which evaluates eight heavily occluded objects. The standard benchmark splits are used for all accuracy evaluations. Section 4.4 further examines sensitivity to stochastic point and model sampling during inference using fixed trained checkpoints.

### 4.2. Evaluation Metrics

To quantitatively evaluate the performance of the RAFusion network, we employ two standard metrics: the Average Distance of Model Points (ADD) and the 2D Reprojection Error [38].

ADD-(S) Metric. This metric measures the average distance between the 3D model points transformed by the ground truth pose and those transformed by the estimated pose. A pose is considered correct if the average distance is less than 10% of the object’s diameter. For asymmetric objects, the ADD metric is calculated as:

(6) $$ADD=\frac{1}{m}\sum_{x\in M}∥\left(Rx+t\right)-\left(\hat{R}x+\hat{t}\right)∥,$$

where *x* denotes a point in the 3D model M, *m* is the total number of points, (R,t) are the ground truth rotation and translation, and $\left(\hat{R},\hat{t}\right)$ are the estimated ones.

For symmetric objects, where multiple poses may be visually indistinguishable, we utilize the ADD-S metric, which computes the average distance to the nearest point in the model:

(7) $$\mathrm{ADD-S}=\frac{1}{m}\sum_{x_{1}\in M}\min_{x_{2}\in M}∥\left(Rx_{1}+t\right)-\left(\hat{R}x_{2}+\hat{t}\right)∥.$$

2D Reprojection Error. This metric assesses pose accuracy by projecting the 3D model points onto the 2D image plane using the estimated pose and comparing them with the projections from the ground truth pose. Following common practice in LINEMOD-based pose evaluation [23,39], a prediction is accepted when the average reprojection error is below 5 pixels. The error is defined as:

(8) $$\mathrm{Proj2D}=\frac{1}{m}\sum_{x\in M}∥\pi \left(K(Rx+t)\right)-\pi \left(K(\hat{R}x+\hat{t})\right)∥,$$

where K represents the camera intrinsic matrix and π(·) denotes the perspective projection operation from homogeneous coordinates to the image plane.

### 4.3. Comparative Experiments

The LINEMOD dataset is divided into a training set (15% of images) and a testing set (85%), following the standard protocol [12]. DenseFusion and RAFusion use identical preprocessing, point sampling, data splits, and iterative-refinement settings. The results for Trans6D+, GS-Pose, PoseMatcher, PVN3D, BB8, and DeepIM are taken from their cited publications. The PointFusion LINEMOD ADD-(S) values follow the reproduced baseline reported by DenseFusion [12]. RGB-based and RGB-D methods are grouped according to their test-time input modalities. Table 1 presents the ADD-(S) results.

RAFusion reaches a mean ADD-(S) accuracy of 97.6%, improving the DenseFusion baseline by 3.3 percentage points and remaining competitive with the other listed methods. The largest gains over DenseFusion occur on challenging categories such as cam and driller, while performance on eggbox and glue is preserved.

As shown in Table 2, RAFusion achieves 97.9% 2D reprojection accuracy, 4.3 percentage points above DenseFusion. Together with the ADD-(S) result, this indicates improved image-plane alignment.

For Occlusion LINEMOD, Table 3 presents a controlled overall comparison between DenseFusion and RAFusion under the same evaluation protocol.

Under the same conditions and evaluation script, RAFusion improves the overall ADD-(S) accuracy by 17.78 percentage points and the 2D reprojection accuracy by 10.52 percentage points compared with DenseFusion.

### 4.4. Robustness and Computational Efficiency

#### 4.4.1. Sensitivity to Stochastic Point Sampling

Random seeds 2026, 2027, and 2028 are used to vary point and model sampling during inference with fixed trained checkpoints. The resulting measurements quantify sensitivity to stochastic point sampling under the same dataset split, target list, and evaluation script. The results are summarized in Table 4.

The small standard deviations indicate low sensitivity to the tested inference sampling seeds under the fixed-checkpoint protocol.

#### 4.4.2. Computational Efficiency

The efficiency profile was measured on an NVIDIA GeForce RTX 4090 GPU (NVIDIA Corporation, Santa Clara, CA, USA) with inference batch size 1 and N=500 sampled points. Latency is the mean time for one PoseNet pose-estimator forward pass; data loading, object segmentation, and iterative refinement are excluded. FPS is calculated as 1000/latency(ms). All four variants use the same hardware, input, and forward-pass settings.

The SE block introduces only a small increase in parameters and FLOPs, whereas the RealFormer module accounts for most of the additional computation. Its self-attention scales quadratically with the number of sampled points. RAFusion records a mean PoseNet forward latency of 2.900 ms and 344.861 FPS under the stated setting. Table 5 reports the complete efficiency comparison.

### 4.5. Ablation Studies

#### 4.5.1. Component Analysis

To verify the effectiveness of each component, ablation studies were conducted on the LINEMOD dataset as summarized in Table 6.

Impact of Adaptive Color Features. Incorporating the SE block into the RGB branch improves accuracy from 94.3% to 95.8%. This gain of 1.5 percentage points indicates that channel-wise recalibration enhances the discriminative power of appearance features by suppressing irrelevant channel noise.

Impact of Global Context Modeling. Integrating the RealFormer module increases accuracy to 96.5%. This improvement of 2.2 percentage points suggests that the residual attention mechanism captures long-range dependencies and geometric relationships more effectively than simple average pooling.

Combined Performance. The full RAFusion network combines both modules and achieves the highest accuracy in the ablation study, 97.6%. The separate improvements of SE and RealFormer, followed by the strongest result when both are enabled, support their complementary roles: SE recalibrates appearance channels before fusion, while RealFormer models cross-point dependencies after fusion.

#### 4.5.2. Hyperparameter Sensitivity

The sensitivity of the model to two key hyperparameters was analyzed: the depth of the RealFormer layers (*L*) and the SE reduction ratio (*r*). The results are presented in Table 7.

Depth of RealFormer Layers. The number of layers determines the capacity for global reasoning. The results show that setting L=2 yields the highest accuracy of 97.6%. A single layer (L=1) improves over the baseline but performs below the two-layer configuration. Increasing the depth to L=4 yields 97.4%, indicating that additional depth does not provide a further gain under the current setting. Therefore, a depth of L=2 is selected.

SE Reduction Ratio. The reduction ratio *r* governs the trade-off between parameter efficiency and representational power. The evaluation indicates that a ratio of r=16 achieves the highest performance of 97.6%. A ratio of r=8 yields 97.5%, while r=32 yields 97.1%, suggesting that stronger channel compression is less suitable under the current configuration. Consequently, r=16 is adopted as the default setting.

### 4.6. Qualitative Visualization

**Figure 4:**
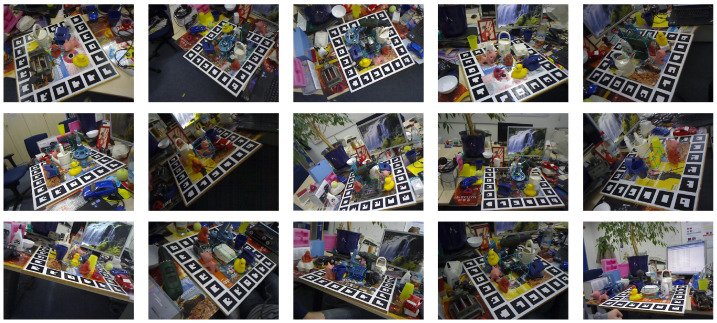
Qualitative results of RAFusion on the LINEMOD dataset. The predictions show close visual alignment across varying viewpoints and cluttered backgrounds. The green bounding boxes represent the ground truth, while the blue bounding boxes represent the pose predictions output by RAFusion.

**Figure 5:**
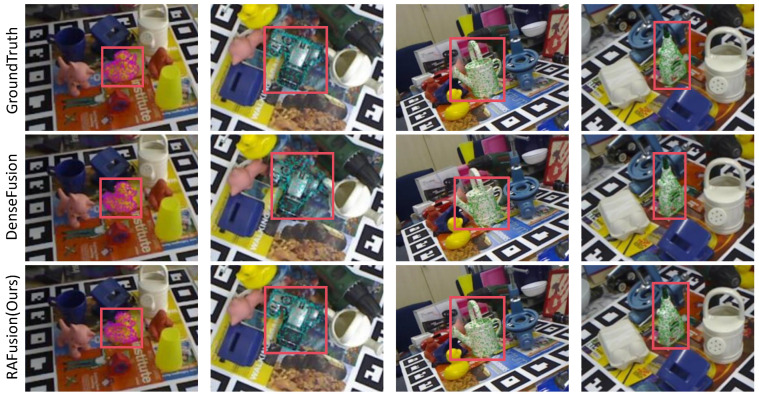
Qualitative comparison of pose estimation results. Each column shows one test example. The first row is the ground-truth pose, the second row is the DenseFusion prediction, and the third row is the RAFusion prediction. Pose accuracy is visualized via projected bounding boxes.

Figure 4 visually illustrates close alignment in the selected cluttered scenes, with predicted bounding boxes largely matching the object contours. Figure 5 compares RAFusion with the baseline: DenseFusion shows visible drift under occlusion, whereas RAFusion produces keypoint projections that are closer to the ground truth in these examples. This visually suggests that integrating residual attention can improve pose recovery in challenging scenarios.

**Figure 6:**
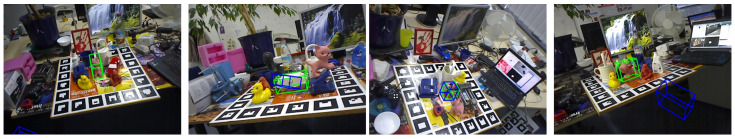
Representative failure cases of RAFusion on Occlusion LINEMOD. The four examples are arranged from left to right. Green and blue boxes denote ground-truth and RAFusion poses, respectively. The cases include severe occlusion and symmetry-induced ambiguity.

Figure 6 shows representative RAFusion failure cases under severe occlusion and symmetry-induced ambiguity.

## 5. Conclusions

This paper presents RAFusion, a DenseFusion-based RGB-D 6D pose estimator that combines channel recalibration before fusion with residual self-attention after fusion. On LINEMOD, the complete model improves mean ADD-(S) from 94.3% to 97.6%. On Occlusion LINEMOD, RAFusion improves ADD-(S) from 40.14% to 57.92% and 2D reprojection accuracy from 17.85% to 28.37%. The ablation study shows that both components contribute to the performance improvement.

RAFusion uses 47.422 M parameters and 26.358 G FLOPs, compared with 21.370 M and 13.488 G for DenseFusion. The pose estimator records a 2.900 ms forward latency on the tested RTX 4090 under the stated forward-pass protocol.

Limitations. Performance remains dependent on the quality of the upstream segmentation mask and the depth measurements. Transparent or reflective surfaces can cause missing depth, while depth noise, the valid operating range of the sensor, and errors in RGB-depth intrinsic or extrinsic calibration can degrade geometric alignment. Severe occlusion and symmetry also remain difficult, as illustrated by the failure cases. Furthermore, the quadratic point-wise attention cost may become more restrictive when the number of sampled points is increased.

Future Work. Future research will investigate more efficient sparse or hierarchical attention, robustness to imperfect segmentation and depth sensing, and stronger supervision for severely occluded objects. ResidualJointAntennaNet [36] also suggests exploring deeper residual structures while controlling computational cost. Render-and-compare optimization or iterative refinement may provide teacher signals for a compact pose regressor, connecting classical pose optimization with learned residual prediction.

## Figures and Tables

**Table 1:** Quantitative comparison on the LINEMOD dataset using the ADD-(S) metric (%). RAFusion achieves the highest reported mean accuracy in this table and improves over the DenseFusion baseline.

	RGB-Based Input			RGB-D Input			
Object	Trans6D+ [40]	GS-Pose [41]	PoseMatcher [42]	PointFusion [12,43]	PVN3D [11]	DenseFusion [12]	RAFusion (Ours)
ape	88.3	71.0	59.2	70.4	95.5	92.3	94.1
benchvise	99.4	99.8	98.1	80.7	94.5	93.2	97.4
cam	97.8	98.2	93.4	60.8	94.2	94.4	98.7
can	99.1	97.7	96.0	61.1	94.3	93.1	97.4
cat	93.2	86.7	88.0	79.1	95.5	96.5	98.0
driller	99.5	96.2	98.4	47.3	93.3	87.0	95.0
duck	87.8	77.2	54.1	63.0	94.6	92.3	96.2
eggbox	100.0	99.6	97.8	99.9	100.0	99.8	100.0
glue	99.8	98.4	91.5	99.3	100.0	100.0	100.0
holepuncher	96.7	98.8	87.9	96.3	95.1	92.1	96.7
iron	99.9	99.6	94.0	97.2	96.3	97.0	99.2
lamp	99.7	98.9	98.1	62.3	93.7	95.3	98.7
phone	99.5	85.0	92.1	78.8	93.6	92.8	97.6
Mean	96.9	92.0	87.5	73.7	95.1	94.3	97.6

**Table 2:** Quantitative comparison on the LINEMOD dataset using the 2D reprojection metric (%, 5-pixel threshold). RAFusion attains the highest reported mean accuracy among the listed RGB-D methods and improves over the DenseFusion baseline by 4.3 percentage points.

	RGB			RGB-D	
Obj.	BB8 [39]	GS-Pose [41]	DIM [7]	DF [12]	RAF
ape	96.6	97.9	98.4	96.9	97.7
benchvise	90.1	98.9	97.0	88.3	94.8
cam	86.0	99.1	98.9	93.8	98.9
can	91.2	97.6	99.7	96.1	98.6
cat	98.8	98.9	98.7	96.1	99.4
driller	80.9	93.7	96.1	84.8	96.8
duck	92.2	97.8	98.5	98.5	98.9
eggbox	91.0	97.1	96.2	99.3	100.0
glue	92.3	97.4	98.9	95.5	99.9
holepuncher	95.3	98.8	96.3	87.9	95.9
iron	84.8	99.6	97.2	94.0	96.3
lamp	75.8	94.2	94.2	92.3	96.9
phone	85.3	93.8	97.7	93.0	98.7
Mean	89.3	97.3	97.5	93.6	97.9

**Table 3:** Controlled overall comparison on the Occlusion LINEMOD dataset. DenseFusion and RAFusion checkpoints are evaluated on the same 1445 target instances using the same local evaluation protocol.

Model	ADD-(S) (%)	2D Reprojection (%)	Targets
DenseFusion	40.14	17.85	1445
RAFusion (ours)	57.92	28.37	1445

**Table 4:** Sensitivity to stochastic point sampling on Occlusion LINEMOD. Using the same fixed checkpoint, values are reported as mean plus or minus standard deviation over inference sampling seeds 2026, 2027, and 2028.

Model	ADD-(S) (%)	2D Reprojection (%)
DenseFusion	40.60±0.42	17.76±0.08
RAFusion (ours)	57.67±0.38	28.47±0.08

**Table 5:** Computational efficiency comparison. FLOPs and latency correspond to one PoseNet estimator forward pass under the same hardware and input settings.

Model	Params (M)	FLOPs (G)	Latency (ms)	FPS
DenseFusion baseline	21.370	13.488	1.780	561.803
DenseFusion + SE	21.457	13.490	1.883	531.171
DenseFusion + RealFormer	47.335	26.356	2.770	361.048
RAFusion	47.422	26.358	2.900	344.861

**Table 6:** Ablation study of different components on the LINEMOD dataset. The results show that both the SE block and the RealFormer module contribute to the performance improvement, with the full RAFusion model achieving the highest accuracy. A checkmark indicates that the corresponding module is included, whereas – indicates that it is not included.

Method	SE	RealFormer	ADD-(S)
Baseline (DenseFusion)	–	–	94.3%
Baseline + SE	✓	–	95.8%
Baseline + RealFormer	–	✓	96.5%
RAFusion (ours)	✓	✓	97.6%

**Table 7:** Hyperparameter sensitivity analysis. Impact of RealFormer depth (*L*) and SE reduction ratio (*r*) on accuracy.

RealFormer Depth *L*		SE Reduction Ratio *r*	
Value	ADD-(S)	Value	ADD-(S)
L=1	96.9%	r=8	97.5%
L=2	97.6%	r=16	97.6%
L=4	97.4%	r=32	97.1%

## Data Availability

The LINEMOD and Occlusion LINEMOD datasets analyzed in this study are publicly available from their original benchmark repositories and are described in the corresponding benchmark publications cited in this manuscript. The source code is available at https://github.com/EstherZhangGit/RAFusion (accessed on 13 July 2026).

## References

[1] Guan J., Hao Y., Wu Q., Li S., Fang Y. (2024). A Survey of 6DoF Object Pose Estimation Methods for Different Application Scenarios. Sensors.

[2] Gorschlüter F., Rojtberg P., Pöllabauer T. (2022). A Survey of 6D Object Detection Based on 3D Models for Industrial Applications. J. Imaging.

[3] Tang C., Zhang M., Zhao Y., Shan S. (2025). Category-Level 6D Pose Estimation Based on Deep Cross-Modal Feature Fusion. Signal Image Video Process..

[4] Zhao Q., Su Y., Zhang H. (2025). STME-Net: Spatio-Temporal Motion Excitation Network for Action Recognition. J. Real-Time Image Process..

[5] Wu F., Liu D., An K., Zhang H. (2024). Image Retrieval Based on Dimensionality Reduction of Second-Order Information. Signal Image Video Process..

[6] Jin M., Li J., Zhang L. (2022). DOPE++: 6D Pose Estimation Algorithm for Weakly Textured Objects Based on Deep Neural Networks. PLoS ONE.

[7] Li Y., Wang G., Ji X., Xiang Y., Fox D. (2018). DeepIM: Deep Iterative Matching for 6D Pose Estimation. Proceedings of the Computer Vision—ECCV 2018.

[8] Song Y., Tang C. (2024). A RGB-D Feature Fusion Network for Occluded Object 6D Pose Estimation. Signal Image Video Process..

[9] Zhang H., Zhao X., Luo R., Wang Z., Wang G., An K. (2026). A Roadmap of Mathematical Optimization for Visual SLAM in Dynamic Environments. Mathematics.

[10] Xiang Y., Schmidt T., Narayanan V., Fox D. PoseCNN: A Convolutional Neural Network for 6D Object Pose Estimation in Cluttered Scenes. Proceedings of the Robotics: Science and Systems XIV.

[11] He Y., Sun W., Huang H., Liu J., Fan H., Sun J. (2020). PVN3D: A Deep Point-wise 3D Keypoints Voting Network for 6DoF Pose Estimation. Proceedings of the IEEE/CVF Conference on Computer Vision and Pattern Recognition.

[12] Wang C., Xu D., Zhu Y., Martin-Martin R., Lu C., Fei-Fei L., Savarese S. (2019). DenseFusion: 6D Object Pose Estimation by Iterative Dense Fusion. Proceedings of the IEEE/CVF Conference on Computer Vision and Pattern Recognition.

[13] Hu J., Shen L., Sun G. (2018). Squeeze-and-Excitation Networks. Proceedings of the IEEE/CVF Conference on Computer Vision and Pattern Recognition.

[14] Vaswani A., Shazeer N., Parmar N., Uszkoreit J., Jones L., Gomez A.N., Kaiser L., Polosukhin I. (2017). Attention Is All You Need. Proceedings of the Advances in Neural Information Processing Systems.

[15] He R., Ravula A., Kanagal B., Ainslie J. (2021). RealFormer: Transformer Likes Residual Attention. Proceedings of the Findings of the Association for Computational Linguistics: ACL-IJCNLP 2021.

[16] Zhang Q., Miao D., Zhang Q., Zhao C., Zhang H., Sun Y., Wang R. (2025). Dynamic Frequency Selection and Spatial Interaction Fusion for Robust Person Search. Inf. Fusion.

[17] Pan S., Wang X. (2021). A Survey on Perspective-n-Point Problem. Proceedings of the 40th Chinese Control Conference.

[18] Tsourounis D., Kastaniotis D., Theoharatos C., Kazantzidis A., Economou G. (2022). SIFT-CNN: When Convolutional Neural Networks Meet Dense SIFT Descriptors for Image and Sequence Classification. J. Imaging.

[19] Gupta S., Thakur K., Kumar M. (2021). 2D-Human Face Recognition Using SIFT and SURF Descriptors of Face’s Feature Regions. Vis. Comput..

[20] Bansal M., Kumar M., Kumar M. (2021). 2D Object Recognition: A Comparative Analysis of SIFT, SURF and ORB Feature Descriptors. Multimed. Tools Appl..

[21] Yi K.M., Trulls E., Lepetit V., Fua P. (2016). LIFT: Learned Invariant Feature Transform. Proceedings of the Computer Vision—ECCV 2016.

[22] Truong P., Apostolopoulos S., Mosinska A., Stucky S., Ciller C., De Zanet S. (2019). GLAMpoints: Greedily Learned Accurate Match Points. Proceedings of the IEEE/CVF International Conference on Computer Vision.

[23] Peng S., Liu Y., Huang Q., Bao H., Zhou X. (2019). PVNet: Pixel-wise Voting Network for 6DoF Pose Estimation. Proceedings of the IEEE/CVF Conference on Computer Vision and Pattern Recognition.

[24] Zakharov S., Shugurov I., Ilic S. (2019). DPOD: 6D Pose Object Detector and Refiner. Proceedings of the IEEE/CVF International Conference on Computer Vision.

[25] Lu W., Wan G., Zhou Y., Fu X., Yuan P., Song S. (2019). DeepVCP: An End-to-End Deep Neural Network for Point Cloud Registration. Proceedings of the IEEE/CVF International Conference on Computer Vision.

[26] Wada K., Sucar E., James S., Lenton D., Davison A.J. (2020). MoreFusion: Multi-object Reasoning for 6D Pose Estimation from Volumetric Fusion. Proceedings of the IEEE/CVF Conference on Computer Vision and Pattern Recognition.

[27] Chen W., Jia X., Chang H.J., Duan J., Leonardis A. (2020). G2L-Net: Global to Local Network for Real-time 6D Pose Estimation with Embedding Vector Features. Proceedings of the IEEE/CVF Conference on Computer Vision and Pattern Recognition.

[28] Wang X., Girshick R., Gupta A., He K. (2018). Non-local Neural Networks. Proceedings of the IEEE/CVF Conference on Computer Vision and Pattern Recognition.

[29] Wang Q., Wu B., Zhu P., Li P., Zuo W., Hu Q. (2020). ECA-Net: Efficient Channel Attention for Deep Convolutional Neural Networks. Proceedings of the IEEE/CVF Conference on Computer Vision and Pattern Recognition.

[30] Woo S., Park J., Lee J.Y., Kweon I.S. (2018). CBAM: Convolutional Block Attention Module. Proceedings of the Computer Vision—ECCV 2018.

[31] Zhang Q., Miao D., Zhang Q., Wang C., Li Y., Zhang H., Zhao C. (2024). Learning Adaptive Shift and Task Decoupling for Discriminative One-step Person Search. Knowl.-Based Syst..

[32] Yew Z.J., Lee G.H. (2018). 3DFeat-Net: Weakly Supervised Local 3D Features for Point Cloud Registration. Proceedings of the Computer Vision—ECCV 2018.

[33] Carion N., Massa F., Synnaeve G., Usunier N., Kirillov A., Zagoruyko S. (2020). End-to-End Object Detection with Transformers. Proceedings of the Computer Vision—ECCV 2020.

[34] Wu X., Jiang L., Wang P.S., Liu Z., Liu X., Qiao Y., Ouyang W., He T., Zhao H. (2024). Point Transformer V3: Simpler, Faster, Stronger. Proceedings of the IEEE/CVF Conference on Computer Vision and Pattern Recognition.

[35] Guo M.H., Cai J.X., Liu Z.N., Mu T.J., Martin R.R., Hu S.M. (2021). PCT: Point Cloud Transformer. Comput. Vis. Media.

[36] Cai J., Qi Y., Liu S., Chen R. (2025). A Residual Joint Antenna Network for Joint Transmit–Receive Antenna Subset Selection in MIMO Systems. IEEE Trans. Antennas Propag..

[37] Liu Y., Ma L., Ren Y., Qi S. (2025). 6-DoF Object Pose Estimation Based on Deep Learning and Iterative Optimization Techniques. Signal Image Video Process..

[38] Hodaň T., Michel F., Brachmann E., Kehl W., Buch A.G., Kraft D., Drost B., Vidal J., Ihrke S., Zabulis X. (2018). BOP: Benchmark for 6D Object Pose Estimation. Proceedings of the Computer Vision—ECCV 2018.

[39] Rad M., Lepetit V. (2017). BB8: A Scalable, Accurate, Robust to Partial Occlusion Method for Predicting the 3D Poses of Challenging Objects without Using Depth. Proceedings of the IEEE International Conference on Computer Vision.

[40] Zhang Z., Chen W., Zheng L., Leonardis A., Chang H.J. (2023). Trans6D: Transformer-Based 6D Object Pose Estimation and Refinement. Proceedings of the Computer Vision—ECCV 2022 Workshops.

[41] Cai D., Heikkilä J., Rahtu E. (2025). GS-Pose: Generalizable Segmentation-Based 6D Object Pose Estimation with 3D Gaussian Splatting. Proceedings of the 2025 International Conference on 3D Vision (3DV).

[42] Castro P., Kim T.K. (2023). PoseMatcher: One-shot 6D Object Pose Estimation by Deep Feature Matching. Proceedings of the IEEE/CVF International Conference on Computer Vision Workshops.

[43] Xu D., Anguelov D., Jain A. (2018). PointFusion: Deep Sensor Fusion for 3D Bounding Box Estimation. Proceedings of the IEEE/CVF Conference on Computer Vision and Pattern Recognition.

